# Hydrate of Chloral

**Published:** 1870-08

**Authors:** 


Hydrate of Chloral.—Dr. J. M. DeCosta contributes to the *Am. Jour. Med. Science* some valuable notes, chiefly climical on the effects of this agent.

Hydrate of Chloral chrystalizes in snow-white needles, has a peculiar, somewhat penetrating odor, and dissolves largely in water without being decomposed. It can be administered only in aqueous solution, but may be given by the stomach, rectum, or hypodermically; its bitter and acrid taste can be disguised by the addition of mucilage or syrup, particularly the syrup of orange peel. It is incompatible with the alkalies. The solution for hypodermic injection should be neutral; but this method had better be avoided, as sometimes fearful phlegmons ensue upon its use; this, however, rarely happens.

The solution of chloral is said to alter by keeping, hence it should be prepared in small quantities.

It is claimed for this new agent, that it produces narcotism with safety, that its hypnotic effects are unattended with restlessness or excitement, that no unpleasant symptoms follows the deep sleep it causes, that it quiets pain, and may remove sensibility completely; also that it lowers the temperature and occasions great muscular relaxation.

The average dose of chloral is from 20 to 40 grains by the mouth, and about 15 hypoderically. It may be given in certain cases in much larger doses, but often causes striking effects in smaller portions. I have seen no *hypnotic effect* from doses less than 20 grains. In that dose it acts fairly in some, while others require several such doses to procure sleep. In one patient not less than 60 grains given in 20 grain doses every hour at bedtime, produced any effect.

The *character of the sleep* is generally sound, but in exceptional cases the patients are troubled with vivid and often unpleasant dreams. Very large doses given at once obviates the disturbance, but prudence forbids their use—owing to individual peculiarity—unless we know how they may effect the patient. When the medicine acts well the sleep is generally induced quickly. I have observed no hyperæsthesia during the sleep, as has been asserted to be the case. Anesthæsia does not follow ordinary doses; it is only the result of very heavy, and, therefore, dangerous doses.

The patient usually awakes naturally, and without mental confusion, yet there may be slight frontal headache, with nausea, and pain at the pit of the stomach. In a few cases I have observed an unsteadiness of gait, resembling that of alcoholic intoxication, and a very free action of the skin; profuse sweating may show itself while the patient is sleeping. The urinary secretion was found augmented under the influence of this drug. The pulse slightly decreases in the number of its beats, and respiration and temperature can only slightly be changed with moderate doses.

We find this sedative of value in restlessness, hysteria, acute mania, and delirium tremens. In the latter affection I have used it in ten cases, and my experience is that it is rather a valuable adjuvant to the treatment than one that can alone overcome the malady. In some cases 90 to 150 grains of chloral were given in the 24 hours.

Chloral relieves pain, and hence is valuable in neuralgias, colic, etc.; in lead colic, especially, it has proved of much service in medium doses. But its action in alleviating pain is not constant, sometimes it acts and sometimes it does not. In coughs, hiccough and chorea, I have been disappointed with it.

Chloral is not without danger, and we should give it intentative doses, carefully watching its effect. It is contraindicated in all enfeebled conditions of the heart. Using opium and chloral together is, I think, often a decided advantage; they do not antagonise but aid each other.

Dr. J. B. Russell, of Glasgow, (*Glasgow Med. Jour.*), has given chloral largely in typhus, as an hypnotic. In 40 grain doses a tonic effect was produced; the heart's action became irregular and slower. In many of his cases there was at first slight stimulant effect, but this was very transient. The chloral acted as a sedative and hypuotic with remarkable uniformity and rapidity, in almost every form of cerebral excitement incident to febrile disease.

1. The chloral sleep, he says, is different from that of opium, and this is more safe than the latter drug. The chloral sleeper may be aroused at any time; he is at once in full command of all his functions, may take food, pass urine, cough, and expectorate; he may then drop over into unconsciousness again at once. Not so after opium has been administered.

2. The excretions are not effected by chloral; they are by opium.

3. Opium is an unsafe medicine for children; chloral is perfectly safe.

4. Opiates, even with tartar emetic, are uncertain; I have met with no patient who resists the action of chloral.

Dr. R., in his doses to children and the young, makes the number of grains about equal the years of the patient, if under twenty-five.

A theory of Leibreich is, that chloral is transformed into chloroform in the blood by its alkali; in typhus, as the blood is excessively alkaline, this drug ought to produce its effects in smaller doses than usual. This Dr. R. observed in every case to be the fact.

Dr. A. M. Adams (*Lancet*), Dr. De la Harpe (*L'Unione Med.*), and Dr. S. Teller (*Med. Record*), all attest the value of chloral in puerperal and acute mania. Especially after it had been found impossible to get sleep from opiates and bromide of potassium was this agent found to give prompt relief.

Dr. Richardson records a case (*Practitioner*) of toxic action of chloral, in which from 40 to 50 grains had induced such a prostration and collapse that death was strongly threatened—cold extremities, juclitation, sense of sinking, gasping breath were they symptoms. He administered stimulants, white of egg, warmth to extremities, and sinipisms to the cardice region, with good effect.

Sir J. Y. Simpson says of chloral: I have found it as a general law as sure a producer of sleep and soother of pain as opium, or any of its preparations. It is usually swifter in the induction of its narcotism, more tranquil in its action, and more prolonged in its effect than opiates, when taken as hypuotics. It is without many of the drawbacks of opium, such as constipation and tendency to excite nausea and vomiting. It will not fulfil all the many indications for which opium is used in medicine, but it will prove a very valuable anodyne in some cases of neuralgia and dysmenorrhœa.—*Med. News, Times and Gazette St. Louis Med. Journal.*

